# The molecular characterization of seven novel GLI family zinc finger 3 (*GLI3*) variants in Chinese families with limb malformations

**DOI:** 10.3389/fgene.2025.1650790

**Published:** 2025-10-09

**Authors:** Siyuan Tao, Xuyu Gu, Xiaodong Wang, Xiaofang Shen, Xiuli Zhao

**Affiliations:** 1 Key Laboratory in Science and Technology Development Project of Suzhou (CN), Pediatric Orthopedics, Children’s Hospital of Soochow University, Suzhou, China; 2 Center for Rare Diseases, State Key Laboratory of Complex, Severe, and Rare Diseases, Peking Union Medical College Hospital, Chinese Academy of Medical Sciences & Peking Union Medical College, Beijing, China; 3 Department of Medical Genetics, State Key Laboratory for Complex, Severe, and Rare Diseases, Institute of Basic Medical Sciences, Chinese Academy of Medical Sciences, School of Basic Medicine, Peking Union Medical College, Beijing, China

**Keywords:** GLI family zinc finger 3, limb malformations, next-generation sequencing, novel variants, polydactyly

## Abstract

**Background:**

GLI family zinc finger 3 (*GLI3*) is a transcription factor involved in limb development. *GLI3* gene variants have been shown to be associated with several human congenital limb malformations, including Greig cephalopolysyndactyly, Pallister–Hall syndrome, non-syndromic postaxial polydactyly (PAP-A/B), and preaxial polydactyly type IV (PPD-IV). The aim of this study was to identify *GLI3* gene variants in ten Chinese families with limb malformations.

**Methods:**

Ten Chinese families with limb malformations were recruited. Variant screening in probands was then performed using NGS, with candidate pathogenic variants verified by polymerase chain reaction (PCR) combined with Sanger DNA sequencing. Variant pathogenicity was evaluated using bioinformatics, evolutionary conservation, and disease and mutant allele co-segregation approaches. The biological effects of missense variants were predicted by three-dimensional protein conformation analysis.

**Results:**

Ten *GLI3* variants were identified: two missense variants c.1063G>A (p.Val355Ile) and c.1489C>A (p.Leu497Ile), four nonsense variants c.2374C>T (p.Arg792*), c.2008C>T (p.Gln670*), c.1096 C>T (p.Arg366*), and c.2029C>T (p.Gln677*); three frameshift variants c.600delC (p.Tyr200*), c.1880_1881del (p.His627Argfs*48), and c.811_812delCT (p.Leu271Serfs*5); a large fragment deletion of NC_000007.14: g.42061081_42069739. Seven of these ten variants have never been recorded in the Human Gene Variant Database.

**Conclusion:**

Ten *GLI3* variants were successfully identified in families with different limb malformations, indicating significant clinical and allelic heterogeneity of *GLI3*-related limb malformations. The present study expands the spectra of pathogenic variants and clinical manifestation for *GLI3*-related morphological disorder and provides solid evidence for genetic counseling and prenatal gene diagnosis in the affected families.

## Introduction

Congenital limb malformations are a heterogeneous group of defects present at birth that primarily affect the morphology and function of the hands and feet of patients. The malformation can be isolated or part of a syndrome and may be caused by genetic or environmental factors (Zguricas et al., 1998). Polydactyly is one of the most common limb malformations in humans. The global incidence is approximately 0.03%–0.36% (Malik, 2014). Most families with polydactyly exhibit autosomal dominant inheritance pattern, while others show autosomal recessive inheritance patterns (Farrugia and Calleja-Agius, 2016). Non-syndromic polydactyly can be categorized according to the location of the finger (toe) deformity as preaxial polydactyly (PPD), postaxial polydactyly (PAP), and central polydactyly, with PPD most common and central polydactyly least common (Fujioka et al., 2005). During human limb formation, embryonic cells initially develop into limb buds, followed by bones, blood vessels, nerves, and muscles, and eventually a hand or foot is generated with a specific shape and number of digits (Bashur et al., 2014). Limb development is a complex process that requires a balance between cell proliferation, cell cycle progression, cell fate determination, and cell differentiation under finely controlled multilayered regulation (Kronenberg, 2003) (Deng et al., 2015). Chromosome 7p has been found to be a hotspot region of variants related to polydactyly (Malik, 2014).

GLI family zinc finger 3 (*GLI3*) is an evolutionarily conserved gene located on human chromosome 7p13 and consists of 14 exons (Zou et al., 2019). Its mRNA is 8.5 kb long, encodes a polypeptide chain of 1580 amino acids containing five highly conserved tandem zinc finger structures with specific DNA sequence affinities (Démurger et al., 2015), and ultimately encodes an important zinc-finger transcription factor implicated in early vertebrate limb development (Kang et al., 1997). The transcription factor is a member of the Hedgehog (Hh/HH) signaling pathway and has important roles in early limb development in animals. Under normal conditions, *GLI3* is processed to the long GLI3-FL peptide form with transcription-promoting properties and has important roles regulating human limb bud development, particularly in the anterior–posterior axis. However, under certain pathological conditions, the gene is processed to the shorter GLI3R peptide form, which exerts inhibitory transcriptional effects (Ruiz i Altaba, 1999). The GLI3 protein binds to specific DNA sequences via its DNA-binding region and regulates the switching on/off of certain genes (Matissek and Elsawa, 2020). Variants located in different GLI3 structural domains can lead to different congenital disorders, including Greig cephalopolysyndactyly syndrome (GCPS; MIM 175700), Pallister–Hall syndrome (PHS; MIM 146510), non-syndromic PAP (PAP-A/B; MIM 174200), and PPD type IV (PPD-IV; MIM 174700) (Al-Qattan et al., 2017).

In this study, we collected over 50 Chinese families with limb malformations. Candidate variants were identified by NGS and verified by Sanger sequencing. We identified ten families with pathogenic variants of *GLI3*, including seven new variants. This study expanded the spectrum of *GLI3* pathogenic variants associated with limb malformations, thereby enhancing our understanding of the genetic underpinnings of such malformations.

## Materials and methods

### Subjects and DNA samples

This study totally collected over 50 families with congenital limb malformations from outpatient clinics between 2023 and 2025. Clinical testing was performed by physical and X-ray examinations before surgical operation. Peripheral blood samples (3–5 mL) were collected from the probands, and all available family members and genomic DNA was extracted using a conventional proteinase K‐phenol‐chloroform method from peripheral blood (Li and Durbin, 2009). Among these, ten families were confirmed to have the variants of *GLI3* gene and were included in this study.

### Next-generation sequencing

Next-generation sequencing (NGS) used in this study included whole-exome sequencing (WES) and whole-genome sequencing (WGS). WES was performed in all probands, and the schedule for WES involved exome capture performed using an Agilent Sure Select Human All Exon kit (Agilent Technologies, Wilmington, DE, United States) and DNA being fragmented into approximately 150–300 base-pair (bp) lengths by ultrasonic treatment. Subsequently, a capture DNA library for sequencing was established using the HiSeq 2000 platform (Illumina Inc., San Diego, CA, United States) to a mean coverage of ×100. Raw data (FASTQ files) were generated using the Illumina base-calling pipeline. For Proband 10 in whom conventional pathogenic variants have been ruled out by WES, WGS was conducted to identify genomic structural variations and deep intronic variations. WGS was conducted thus: genomic DNA was randomly sheared to approximately 350 bp using a Covaris shearing instrument. The sheared DNA underwent end repair and end blunting to ensure 5′-end phosphorylation. The repaired products were purified using Agencourt SPRIselect beads and size-selected with the Agencourt SPRIselect nucleic acid fragment selection kit to enrich fragments of the target length. The purified, end-blunted DNA fragments underwent A-tailing to add an A base at the 3′end, enabling ligation with the T-overhang of the sequencing adapter. The completed library, after adapter ligation, underwent PCR amplification for enrichment followed by quality control. Based on effective library concentration and data volume requirements, final sequencing was performed on the Illumina NovaSeq6000 platform.

The raw data obtained from next-generation sequencing were aligned to the human reference genome (hg19) using Burrows–Wheeler Aligner backtrack software (Li and Durbin, 2009). File sorting and duplicate marking were performed with the Sequence Alignment/Map (SAM) tools (Li et al., 2009) and Picard (http://broadinstitute.github.io/picard), respectively. Variant detection was conducted using the SAM tools “mpileup” command and BCF tools (http://samtools.github.io/bcftools/bcftools.html), which identified single-nucleotide polymorphisms (SNPs) and insertion–deletion variants (indels). Annotation of variant positions, types, conservation predictions, and other relevant information was performed through ANNOVAR software (Wang et al., 2010) by integrating multiple databases, including the SNP database (dbSNP), 1000 Genomes Project, the Exome Aggregation Consortium (ExAC; http://exac.broadinstitute.org/), and the Human Gene Variant Database (HGMD; http://www.hgmd.cf.ac.uk/ac/index.php). Based on the filtering strategy, synonymous single-nucleotide variants with a minor allele frequency (MAF) > 1% in the 1000 Genomes database were excluded from the candidate pathogenic variants.

### PCR and Sanger sequencing

PCR-Sanger sequencing was used for validation of candidate variants revealed by NGS and family co-segregation of the phenotype and genotype. Genomic DNA/DNA (cDNA) and protein reference sequences of *GLI3* (hg19: NM_000168.6/NC_000007.14 and NP_000159.3) were obtained from the University of California, Santa Cruz (UCSC) Genome Browser database (http://genome. ucsc. edu/) and the National Center for Biotechnology Information (NCBI) Reference Sequence Project. Primers were designed via the Primer3 online tool (http://primer3.ut.ee/) and were then inspected using the UCSC Genome Browser BLAT and *in silico* PCR online tools (Table 1). TaKaRa LA Taq^®^ with GC Buffer (TaKaRa, Shiga, Japan) was used in all PCR volumes, with initial denaturation at 95 C for 3 min, followed by 35 cycles of 94 C for 30 s, 58 C for 40 s, and 72 C for 40 s, with a final extension at 72 C for 8 min. Sequencing results from Applied Biosystems 3730 × l DNA Analyzer (Thermo Fisher Scientific, Waltham, MA, United States) were aligned to reference sequence using Codon Code Aligner (v.6.0.2.6; Codon Code, Centerville, MA, United States).

**TABLE 1:** Primers for PCR and sanger sequencing in pathogenic variant validation.

Proband	Mutation	Forward primer(5’–3’)	Reverse primer(5’–3’)	Product length(bp)
1	c.1063G>A	CTG​CGT​GTA​TGT​GTG​CAC​AC	ACA​GAG​GTG​CCG​TGT​TGA​TT	520 bp
2	c.1880_1881del	GAC​TTT​TGG​GCT​GGG​GCA​TA	CAA​TAC​GGG​TCA​CTG​CCC​TC	503 bp
3	c.2374C>T	AAA​GTG​GCC​AGC​TCC​ATT​CA	CCT​GAG​CAG​ATG​CAT​GGT​CT	513 bp
4	c.2029C>T	AAA​GGA​CTT​TTG​GGC​TGG​G	TGT​GAA​GTC​AGA​AGG​AGA​GTG​A	393 bp
5	c.2008C>T	GAC​TTT​TGG​GCT​GGG​GCA​TA	CAA​TAC​GGG​TCA​CTG​CCC​TC	503 bp
6	c.811_812delCT	AGG​AAT​TGC​TGA​TGT​GGG​TTG	GTT​GCC​TTT​GCC​ATT​TCC​CA	301 bp
7	c.1489C>A	CTT​CAC​AAA​ACC​CTA​GAC​CCA	ATA​AAG​CCC​TCT​CCA​GTT​CG	388 bp
8	c.1096C>T	TGA​GCT​CAG​CGT​TTA​AGT​GA	ATC​GAC​CTG​TCC​CTC​TCA​C	283 bp
9	c.600delC	TTC​CAC​AAG​GCT​CCT​TTG​AA	ACC​ACT​GCC​AAT​GAG​GTG​TT	504 bp
10	g.42061081_42069739del	AGA​ACG​ACT​TTT​GCA​GGT​GT	AGT​TGG​TAG​TTT​TAA​GCA​GCC​T	8941 bp

### Real-time quantitative PCR and Gap-PCR

The large fragment deletion (Family 10) found by WGS was verified by real-time quantitative PCR (qPCR) and Gap-PCR. qPCR was performed in a saturated fluorescence dye method using TB green (TaKaRa, Dalian, China) in a 96-well Thermal iCycler (Bio-Rad, Hercules, CA, United States). The qPCR condition was as follows: initial denaturation at 95 C for 30 s, followed by 39 cycles of 95 C for 5 s (denaturation), 58 C for 30 s (annealing), and 72 C for 5 s (extension, optimized for Taq enzyme activity). Subsequently, a melting curve analysis was conducted to assess amplification specificity: 60 C for 1 min, then 60–95 C on 0.5 C per cycle (continuous fluorescence collection), and final hold at 40 C for 30 s. Based on gross deletion detected by qPCR, the breakpoint was identified by Gap-PCR combined by a 2% agarose gel electrophoresis and DNA sanger sequencing. The primers used for qPCR and Gap-PCR are listed in Table 2.

**TABLE 2:** Primer sequences utilized in qPCR and Gap-PCR for large fragment deletion.

Targrt region	Forward primer(5’–3’)	Reverse primer(5’–3’)	Product length
Exon 6	CCG​AAA​ACG​TAC​ACT​GTC​CA	GTG​ACC​ATA​GGA​GCC​ACT​TG	142 bp
Exon 7	CGT​CTC​TCT​CCA​CAT​GCA​TC	AGG​CCT​CTG​TGT​TGG​AAA​AG	115 bp
Exon 8	GCA​AAT​GTG​ACC​AGC​AGA​AC	GGA​GGT​CTT​CAT​CGG​GTT​TG	105 bp
Exon 9	CCC​CAC​CCT​CTT​CTT​TCA​GG	GCT​CTT​GGG​TGT​CGA​ACT​C	149 bp
Exon 10	GAA​ACC​CTT​CAA​AGC​CCA​GT	CCA​CCC​ACT​TCT​GTA​CTC​AC	96 bp
g.42061081_42069739	AGA​ACG​ACT​TTT​GCA​GGT​GT	TGG​TGG​TCA​TTT​AGT​TGG​TAG​T	8941 bp

### Bioinformatics

The VarCards2 (https://genemed.tech/varcards2) platform was used to predict the potential impact of all candidate variants using more than 30 algorithms, including the tools of allele frequency, pathogenicity prediction scores, and databases of disease variants. Variant pathogenicity was assessed based on ACMG guidelines. For the missense variants, evolutionary conservation across multiple species was analyzed with MEGA-11 software (Molecular Evolutionary Genetics Analysis software); the spatial structure of the GLI3 protein was predicted with Swiss model by comparing wild-type and mutant molecules, and the three-dimensional structure of the proteins was displayed in Swiss-Pdb Viewer software. For frameshift variants, DNAMAN (version 5.2.2; Lynnon Bio soft) was used to predict the possible protein truncation.

## Results

### Clinical evaluation

**FIGURE 1:**
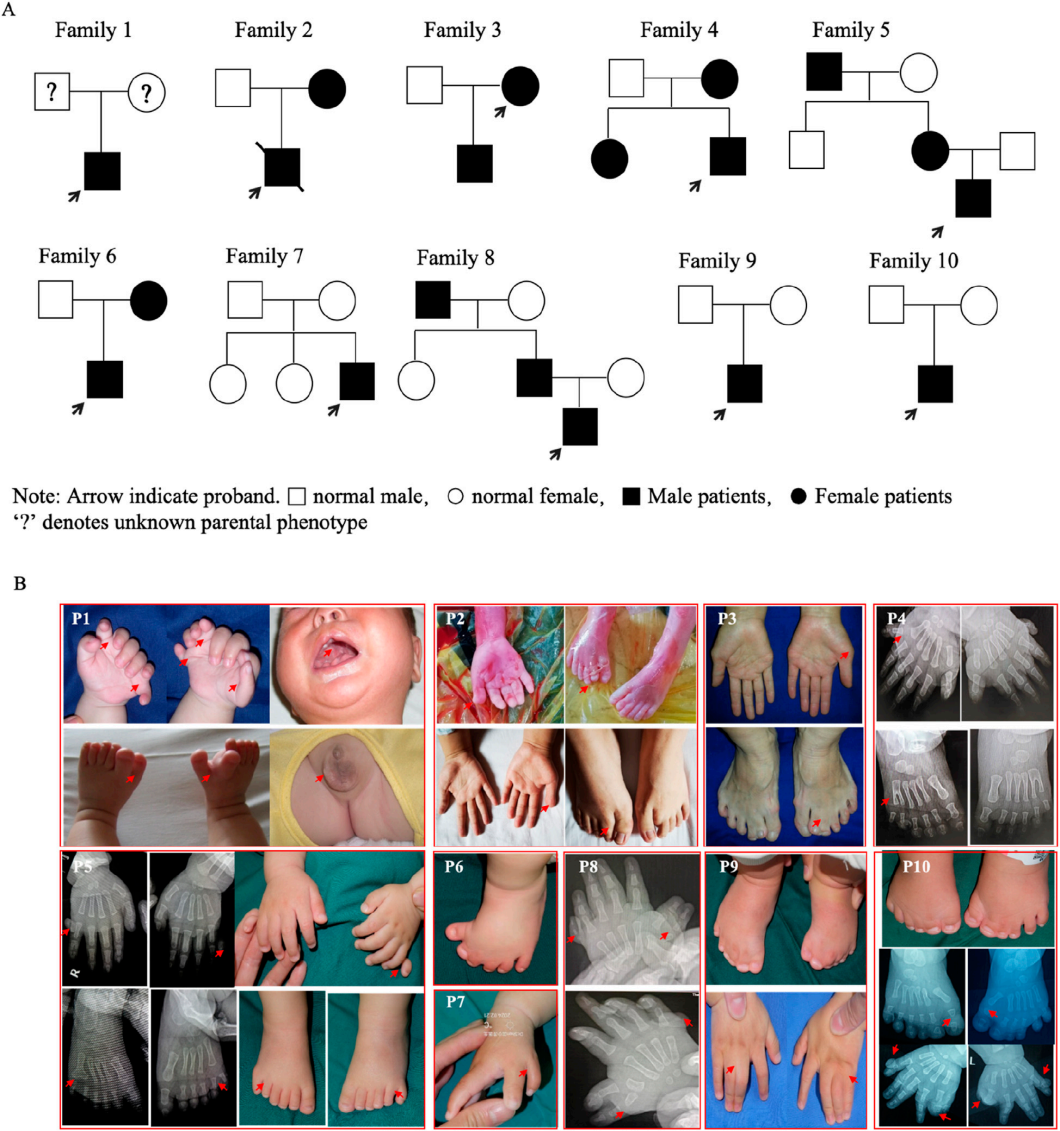
Pedigrees and clinical manifestations of the ten probands. **(A)** Pedigrees of the ten families (Proband 1 was an abandoned infant). **(B)** Clinical manifestations in the probands and some affected parents.

Ten families had limb malformations caused by *GLI3* variants. The pedigrees and clinical manifestations are presented in Figure 1 and Table 3. Based on whether there are disease manifestations beyond limb malformations, these patients were classified into two categories: syndromic and non-syndromic. Proband 1 was an abandoned infant diagnosed with Pallister–Hall syndrome (PHS), presenting with bilateral polydactyly, cryptorchidism, and an incomplete cleft lip/palate. The remaining nine probands displayed non-syndromic limb malformations. Proband 2 was a fetus of induced termination, with a unilateral broad thumb and bilateral central polydactyly of the feet and displayed clinical manifestations akin to those of his mother (blood sample not available). Proband 3 presented with bilateral preaxial polydactyly of the hands (surgically excised) and syndactyly of the second and third toes. Probands 4 and 5 had postaxial polydactyly (PAP-A/B). Proband 6 exhibited complex polydactyly–syndactyly solely in both feet, but his mother exhibited brachydactyly of both thumbs and syndactyly between the third and fourth fingers on her left hand (preoperative photographs not available). Probands 7 and 8 both presented with Wassel type V preaxial polydactyly (PPD-IV), but Proband 8 also presented with bilateral postaxial polydactyly. Isolated PPD-IV is often categorized within the GCPS spectrum as a subtype characterized by mild craniofacial features that can be difficult to distinguish from unaffected individuals. Probands 9 and 10 displayed foot characteristics similar to those of Proband 6, characterized by complex polysyndactyly, but Proband 9 exhibited syndactyly in both hands without polydactyly, whereas Proband 10 showed both preaxial polysyndactyly and postaxial polydactyly in both hands.

**TABLE 3:** Summary of clinical symptoms and pathogenic *GLI3* variants found in families with limb malformations.

Proband	Age	Clinical symptoms	Variant type	Nucleotide change	Amino acid change	ACMG Criteria	Pathogenicity	Novel or reported
1	8 months	Pallister–Hall syndrome; central polydactyly of both hands; preaxial polydactyly of both feet; micropenis; incomplete cleft lip; high palate.	Missense	c.1063G>A	p.Val355Ile	PM2+PP1+PP3	Likely Pathogenic	Novel
2	Fetus	Broad thumb on one side of the hand; preaxial polydactyly on both feet.	Frameshift	c.1880_1881del	p.His627Argfs*48	PVS1+ PM2+PS4+PP1	Pathogenic	Reported
3	54 years	Bilateral polydactyly of the hands (surgically removed); bilateral polydactyly of the feet with syndactyly of the second and third toes.	Nonsense	c.2374C>T	p.Arg792*	PVS1+ PM2+PS4+PP1	Pathogenic	Reported
4	6 months	Postaxial polydactyly of both hands and feet.	Nonsense	c.2029C>T	p.Gln677*	PVS1+ PM2+PS4+PP1	Pathogenic	Novel
5	9 months	Postaxial polydactyly of both hands; floating ulnar polydactyly of the little fingers; postaxial hexadactyly deformity of both feet.	Nonsense	c.2008C>T	p.Gln670*	PVS1+PM2+PS4	Pathogenic	Novel
6	8 months	Syndactyly and polydactyly of both feet; multiple *cafe-au-lait* spots on the back and abdomen.	Frameshift	c.811_812delCT	p.Leu271Serfs*5	PVS1+PM2+PP1	Pathogenic	Novel
7	1 year	Wassel type V accessory thumb on the right hand.	Missense	c.1489C>A	p.Leu497Ile	PM2+PP3	Likely Pathogenic	Novel
8	7 months	Polydactyly of both hands; syndactyly of the middle ring fingers; polydactyly of both feet.	Nonsense	c.1096C>T	p.Arg366*	PVS1+PM2+PS4+PP1	Pathogenic	Reported
9	2 years	Synpodactyly of both hands, polydactyly with deformity of both feet accompanied by functional impairment.	Frameshift	c.600delC	p.Tyr200*	PVS1+PM2+PS4	Pathogenic	Novel
10	6 months	Polydactyly of both hands; syndactyly between the third and fourth digits of both hands; polydactyly between the second and third toes.	Large fragment deletion	g.42061081_42069739del	p.?	PVS1+PM2+PS4	Pathogenic	Novel

### Variant identification

**FIGURE 2:**
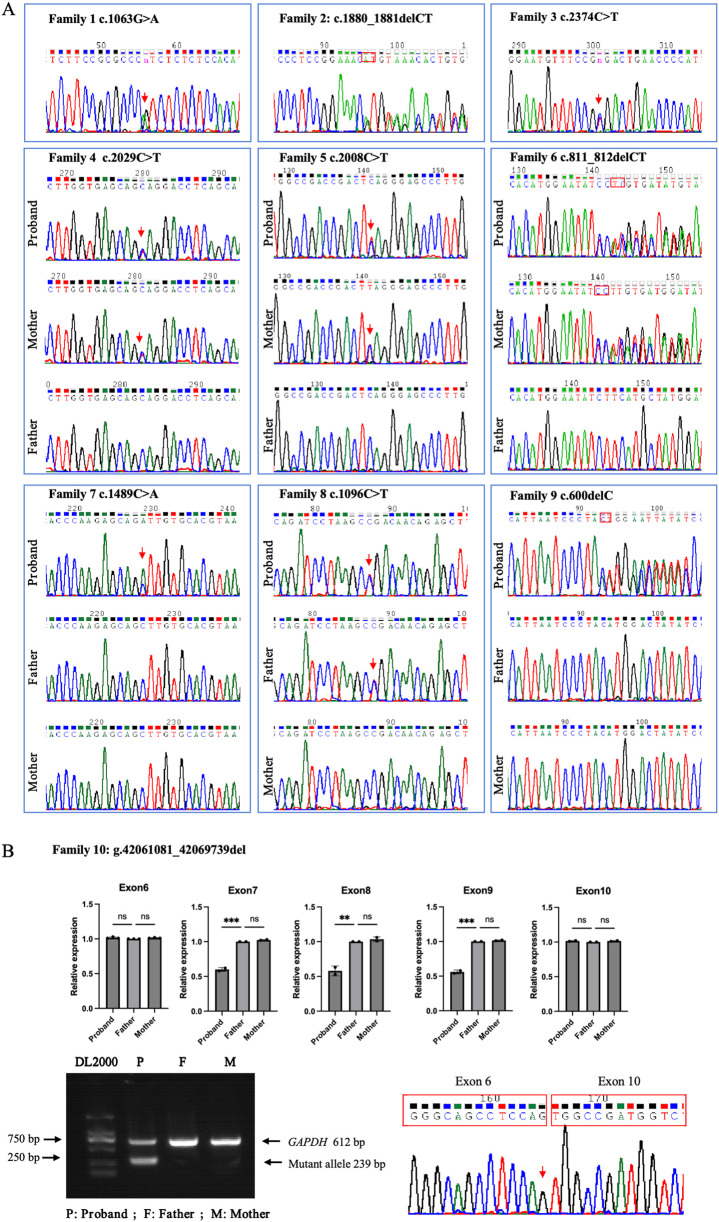
*GLI3* variants identified in the ten families with limb malformations. **(A)** Sanger sequencing results for Families 1–9. **(B)** Large fragment deletion (NC_000007.14: g.42061081_42069739del) of *GLI3* validated in Proband 10 by qPCR, gap-PCR, and Sanger DNA sequencing.

Ten heterozygous *GLI3* variants were screened by NGS combined with Sanger DNA sequencing. These variants included two missense (c.1063G>A; c.1489C>A), four nonsense (c.2374C>T; c.2008C>T; c.1096C>T; c.2029C>T), three frameshift (c.600delC; c.1880_1881del; c.811_812delCT) (Figure 2A), and one gross deletion (NC_000007.14: g.42061081_42069739del), and each patient was a heterozygote of one pathogenic *GLI3* variant (Table 3). The familial samples of Probands 1, 2, and 3 were not available, so the co-segregation of pathogenic variant genotypes and phenotypes cannot be established in these families. In the other seven pedigrees, co-segregation of genotype and phenotype was observed. The pathogenic alleles of Probands 4, 5, and 6 were inherited from their mothers, who exhibited hand and foot malformations. The pathogenic allele in Proband 8 originated from his father, who also showed hand and foot malformations. Probands 7, 9, and 10 were sporadic cases and caused by *de novo* variants (c.1489C>A, c.600delC, g.42061081_42069739del, respectively). qPCR was employed to compare the copy numbers of exons 6-10 of *GLI3* between Proband 10 and his normal parents. The results showed that the dosage of exons 7–9 in Proband 10 was merely half of that in normal parents. Gap-PCR was utilized to pinpoint the breakpoint of the gross deletion, uncovering a deletion of 8,658 bp in this patient. Gap-PCR can specifically amplify a 239 bp DNA fragment in cases of gross deletion in *GLI3*, whereas no such fragment was observed in his parents (Figure 2B). In the ten variants identified above, three (c.1880_1881del, c.2374C>T, and c.1096C>T) had been recorded by HGMD (Professional 2024.4), while the other seven (c.1063G>A, c.600delC, c.2029C>T, c.1489C>A, c.2008C>T, c.811_812delCT, NC_000007.14: g.42061081_42069739del) were novel and reported for the first time.

### Pathogenicity evaluation of candidate variants according to ACMG guidelines

The evidence codes applied and the final pathogenicity classification for each variant are summarized in Table 3. According to database gnomAD v2.1.1, *GLI3* is intolerant of protein-truncating variations (pLI = 1.00, oe = 0.09), indicating that loss of function is a pathogenic mechanism of the truncating variants of *GLI3*. Briefly, null variants (nonsense, frameshift, large deletion) were classified as pathogenic or likely-pathogenic, supported by very strong (PVS1) and supporting (PS4, PM2, and PP1) evidence. The two missense variants were assessed using computational prediction (PP3), allele frequency (PM2), and segregation (PP1) data, resulting in a classification of likely pathogenic.

### *In silico* predictive analysis

**FIGURE 3:**
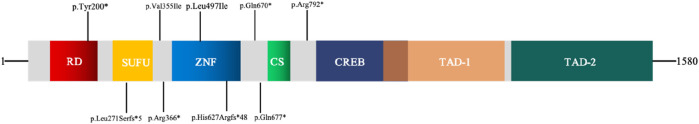
Schematic diagram of transcription factor GLI3. RD: repressor domain; SUFU: SUFU-binding sequence; ZNF: zinc finger domain; CS: cleavage site; CREB: CREB-binding protein; TAD: transcriptional activation domain.

GLI3 is a transcriptional activator, its structure is shown in Figure 3. Variant p. Tyr200* located within the repressor domain is predicted to result in a truncated protein lacking functional repressor and subsequent domains, including the zinc finger motif, proteolytic cleavage site, and activation domain, thereby impairing transcriptional repression. The p. Leu271Serfs*5 variant occurs within the SUFU-binding sequence (SUFU) and is likely to disrupt *GLI3*–SUFU interaction and subsequent processing. Variants p. Val355Ile and p. Arg366*, situated near the junction between the SUFU-binding sequence and the zinc finger domain, may affect repressor function. Variants p. Leu497Ile and p. His627Argfs*48 within the zinc finger domain are expected to impair DNA binding and transcriptional regulation. Variants p. Gln670* and p. Gln677 located close to the proteolytic cleavage site may interfere with normal protein processing. The p. Arg792 variant lies between the cleavage site and the CREB-binding domain and may reduce CBP-binding affinity. The large deletion (NC_000007.14: g.42061081_42069739del) is predicted to cause haploinsufficiency through protein truncation.

**FIGURE 4:**
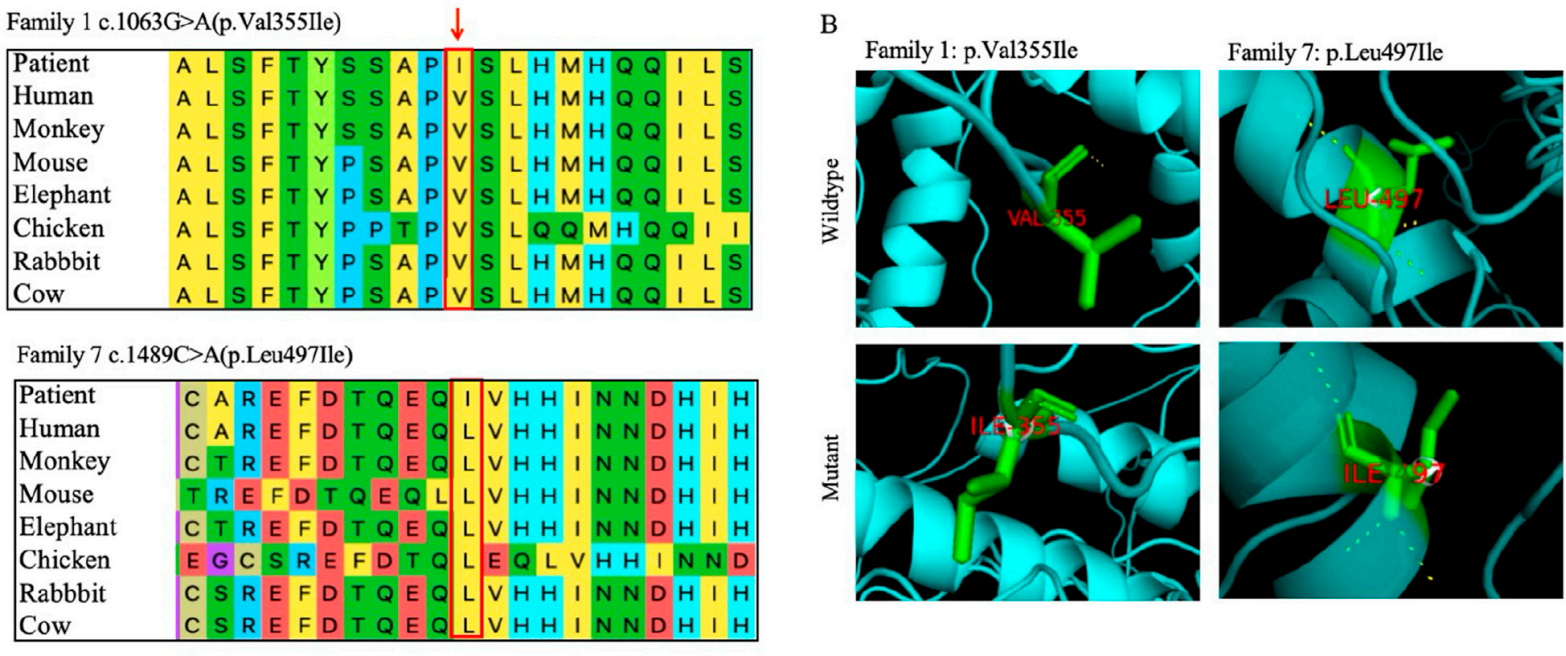
Bioinformatics analysis of two novel missense variants of *GLI3*. **(A)** Evolutionary conservation of amino acids at variant sites. Variant sites marked with red boxes. **(B)** Three-dimensional structural comparisons between mutant and wild types. Three-dimensional changes caused by the variants pVal355Ile and p. Leu497Ile.

Protein conservation analysis and a three-dimensional structure prediction were also conducted for the two missense variants (Figure 4). The conservation analysis of variant sites using multiple sequence alignments indicated that *GLI3* variants c.1063G>A (p.Val355Ile) and c.1489C>A (p.Leu497Ile) were highly conserved across different species (monkey, mouse, elephant, chicken, rabbit, and cow). The two missense variants were analyzed by three protein deleteriousness prediction software (Table 4). The CADD scores for the two missense variants are 22.4 and 25.2, indicating relatively strong predictive scores for harmfulness. Variants c.1063G>A (p.Val355Ile) and c.1489C>A (p. Leu497Ile) were predicted to be potentially deleterious and disease-causing (Polyphen-2 and Variant Taster, respectively). Variant p. Val355Ile may disrupt the packing of the local hydrophobic core by introducing a larger side chain, indirectly affecting the hydrogen bonding network and structural stability. Variant p. Leu497Ile induces alterations in the α-helices, side chains, and hydrogen bonds compared with normal condition, directly interfering with the spatial conformation of the GLI3 protein and decreasing the affinity of its ZNF-domain-binding DNA.

**TABLE 4:** Predicting the functional deleteriousness of missense variants in the GLI3 protein.

Proband	Nucleotide change	Amino acid changes	CADD	Polyphen 2	Mutation taster
1	c.1063G>A	p.Val355Ile	22.4	Possibly damaging	Disease causing
7	c.1489C>A	p.Leu497Ile	25.2	Possibly damaging	Disease causing

## Discussion

Congenital limb deformities are the most common birth defects in newborns (Biesecker, 2002). Around the fourth week of embryonic development, the ventrolateral wall of the embryo begins to form the limb bud of the upper limb and gradually grows to form the ectodermal ridge. Subsequently, the hand plate appears and develops into finger plates. As interdigital cell apoptosis occurs around the eighth week, each finger begins to separate and mature. In this process, various growth and transcription factors and related proteins are involved in joint regulation (Tonkin, 2009). *GLI3* contains zinc finger structures and plays a key role in regulating the Sonic Hedgehog signaling pathway, which is involved in many physiological processes, including cell growth, differentiation, and tissue morphogenesis. *GLI3* has several highly conserved functional domains, including a repressor domain (amino acids (AA) 106–163), a zinc finger binding domain (AA 462–645), a protein hydrolysis cleavage site (AA 703–740), a CREB-binding protein domain (AA 827–1131), a trans-activation domain 2 (AA 1044–1322), and a trans-activation domain 1 (AA 1376–1580) (Ito et al., 2018). Abnormalities in *GLI3* function are associated with the occurrence and development of various developmental diseases and cancers (Tickle and Towers, 2017).

In this study, we recruited over 50 Chinese families with congenital limb malformations. Among them, pathogenic *GLI3* variants were identified in ten families, accounting for approximately 20% of the study cohort. Within these ten confirmed variants, the mutational spectrum consisted of two missense variants and eight protein-truncating variants (including nonsense, frameshift, and a large fragment deletion). According to the genotype–phenotype association model proposed by Johnston et al. (2005), *GLI3*-related phenotypes are primarily determined by the type of variants and their location within the protein. Their study demonstrates that protein-truncating variants (nonsense or frameshift) occurring in the middle third of the gene (amino acid region 666–1161) are generally associated with PHS. In contrast, truncating variants outside this region, missense variants, large deletions, and other variant types predominantly lead to GCPS or related phenotypes. In our study, the mutations identified in Probands 3–5 were located within the amino acid region 666–1161. Unlike the Johnston model, which associates mutations in this region with PHS, these probands exhibited non-syndromic polydactyly. With the exception of Proband 10, who carried a large fragment deletion, the mutations in the remained six probands were located within the amino acid region 1–666. Diverging from the Johnston model, which predicts GCPS for mutations in this region, Proband 1 presented with PHS, while the others manifested non-syndromic polysyndactyly. These observations underscore the remarkable complexity of GLI3-related disorders and further refine the genotype–phenotype model proposed by Johnston et al. (2005).

The clinical phenotypes of patients with *GLI3* variant-induced limb malformations are complex and varied, including PPD, PAP, syndactyly, and other syndromes. No single predominant hotspot variant has been identified in *GLI3*; rather, variants are distributed across the gene. This widespread distribution of variants, affecting various functional domains of the GLI3 protein, is a key contributor to the extensive clinical heterogeneity observed in patients. Clinical diagnoses are highly dependent on high-throughput sequencing. This clinical heterogeneity was evident in our cohort. We observed a spectrum of limb malformations caused by various *GLI3* variants. Furthermore, even the same variant could lead to different presentations. In the c.2374C>T (p.Arg792*) variant reported by Kalff-Suske et al. (1999), their patient had GCPS with clinical PPD manifestations as well as syndactyly of the hands and feet, macrocephaly, a broad nasal root with mild hypertelorism, and a prominent forehead. Proband 3 in our study also had this variant but clinically presented with non-syndromic PAP of both hands and feet. In the c.1096 C>T (p. Arg366*) variant reported by Debeer et al. (2003), their patient had GCPS with bilateral polydactyly of both hands and feet, a broad nose, wide spacing, forehead bulge, and a high and wide forehead. In our study, pedigree 8 also had this variant but clinically presented as non-syndromic polydactyly. In variant c.1880_1881del (p. His627Argfs*48) reported by Johnston et al. (2005), their patient also had GCPS with bilateral polydactyly of both hands and feet, macrocephaly, and hypertelorism. In our study, pedigree 2 also had this variant but clinically presented with non-syndromic bilateral polydactyly. Among the ten variants identified in this study, the majority were concentrated in the N-terminal two-thirds of the protein. A summary of the literature (Wang et al., 2024) shows that the variant sites in patients with polydactyly caused by *GLI3* variants involve each domain of *GLI3*. Therefore, it is speculated that the non-syndromic *GLI3* variants reported to date are distributed across the entire gene. The discovery of additional non-syndromic *GLI3* variants will help us further understand this association.

## Conclusion

Seven novel *GLI3* variants and three previously reported *GLI3* variants were identified in ten Chinese families with polydactyly. Our work significantly expanded the spectra of variants and phenotypes of *GLI3*-related disorders. These findings provide solid evidence for precise diagnosis and genetic counseling for the related families with limb malformations.

## Data Availability

The datasets presented in this study can be found in online repositories. The names of the repository/repositories and accession number(s) can be found in the article/supplementary material.
